# The increased lateral tibial slope may result in inferior long-term clinical outcome after DB-ACL reconstruction

**DOI:** 10.1007/s00402-023-05114-3

**Published:** 2023-11-14

**Authors:** Cheng-Pang Yang, Shih-Feng Hung, Keng-Yi Lin, Yu-Chieh Hung, Yi-Jou Chen, Shang-Yu Yao, Chih-Hao Chiu, Yi-Sheng Chan

**Affiliations:** 1 Department of Orthopedic Surgery, Keelung Chang Gung Memorial Hospital, No. 222, Mai-Chin Rd, Keelung City, 204 Taiwan; 2 Comprehensive Sports Medicine Center, Taoyuan Chang Gung Memorial Hospital, Taoyuan City, 333 Taiwan; 3 Department of Orthopedic Surgery, Linkou Chang Gung Memorial Hospital, Taoyuan City, 333 Taiwan; 4 Department of Orthopedic Surgery, Taoyuan General Hospital, Ministry of Health and Welfare, Taoyuan City, 333 Taiwan

**Keywords:** Double bundle ACL reconstruction, Lateral tibial slope, Long term PROMs results

## Abstract

**Purpose:**

To determine if there is a correlation between lateral tibial slope and long-term clinical results in patients who underwent double-bundle ACL reconstruction.

**Methods:**

We retrospectively reviewed patients that received double-bundle ACL reconstruction at a single institution by a single surgeon from January 2011 to December 2014. All the magnetic resonance imaging were reviewed and lateral tibial slopes (LTS) were recorded by an experienced surgeon and rechecked by the other two authors of this study that specialized in orthopedic knee surgery. The relationship between PROMs measurement and lateral tibial slope were analyzed. The patients were then separated into two groups (LTS > 7.4° and < 7.4°) according to the previous study.

**Results:**

A total of 119 patients were enrolled in this study. All enrolled patients were followed for at least 8 years. The PROMS result were negatively correlated with the lateral tibial slope (*p* values all < 0.001). The patients with high lateral tibial slope had significantly lower PROMS values (Lysholm 94.26 ± 5.61 vs 80.15 ± 8.28, *p* = 0.013; IKDC 82.99 ± 4.55 vs 70.09 ± 7.15, *p* = 0.003; Tegner 9.32 ± 0.95 vs 6.85 ± 1.99, *p* < 0.001). Finally, the LTS cutoff value between patients with “Good” and “Fair” Lysholm score in our study was 7.55 degrees.

**Conclusions:**

Patients with high lateral tibial slope may result in inferior long-term subjective outcomes. The using of double-bundle ACL reconstruction along cannot overcome the negative impact caused by steep lateral tibial slope. A lateral tibial slope of 7.55° may be used as a cut-off for a good clinical outcome.

**Level of evidence:**

III retrospective comparative prognostic trial.

## Introduction

Anterior cruciate ligament (ACL) reconstruction has been one of the standard treatments for ACL rupture [22, 37–39]. After ACL reconstruction, it is expected that patients should be able to play sports at preinjury levels of performance. The results of the previous study indicate that 61% of patients who return to sports after ACL reconstruction surgery self-report that they can play at their preinjury levels of performance by 2–4 years [38].

On the other hand, both biomechanical and clinical studies have shown the negative effect of a steep tibial slope [11, 18]. Patients with a high tibial slope had a higher residual pivot-shift and tibial anterior translation. A steep posterior tibial slope is a common finding among patients who experience multiple ACL failures [5, 15, 16, 27]. In the typical scenario of ACL injury, the lateral femoral condyle subluxates posteriorly on the lateral tibial plateau. According to previous study, lateral-to-medial slope asymmetry, specifically a flat medial tibial slope (MTS) and a steep lateral tibial slope (LTS), may lead to a greater external rotation of the femur and greater internal rotation of the tibia [13]. The consequences of the ACL-injury include unstable knee joint, damage to surrounding soft tissues, and associated pain. A recent study by Grassi et al. suggested that a LTS greater than 7.4° was shown to provide highest sensitivity for ACL failure after primary reconstruction [16]. These studies mostly focused on the graft failure rate and residual pivot shift. However, the correlation of tibial slope and patient subjective outcomes is still unclear.

The aim of this study was to find if there is a correlation between tibial slope and long-term clinical results in patients who underwent double-bundle ACL reconstruction. We hypothesized that double-bundle ACL reconstruction could eliminate the negative effect of a steep posterior slope.

## Methods

### Study population and surgical techniques

This study was approved by the Biomedical Institutional Review Board, and the requirement for informed consent was waived due to the retrospective nature of the study. We reviewed a total of 154 patients who underwent double-bundle ACL reconstruction at a single institution by a single surgeon from January 2011 to December 2014. All patients were followed for at least 8 years. All patients underwent complete radiographic analysis, including knee MRI, and underwent clinical evaluations before surgery. Inclusion criteria included patients that received primary double-bundle ACL reconstruction using hamstring tendon and bio-absorbable screws with hamstring by the same orthopedic surgeon and had a minimum of 8 years of follow-up. Exclusion criteria included incomplete radiographic records, loss to follow-up, previous knee surgery history or revision, pre-existing knee condition, and malignant bone tumor. Demographic data are listed in Table 1.

**Table 1:** Demographic parameters

Numbers	119
Sex (m: f)	113:6
Age at surgery (years)	32.53 ± 7.61
Age at last F/U (years)	44.33 ± 8.34
BMI (kg/m^2^)	26.38 ± 4.26
Mean LTS	6.56° ± 3.38°
Meniscus	
Intact	52
Injured	67
Medial cartilage	
Intact	108
Injured	11
Lateral cartilage	
Intact	115
Injured	4
F/U (month)	144

Values are the means ± standard deviations

*BMI* body mass index at surgery, *LTS* lateral tibial slope, *MTS* medial tibial slope, *F/U* average follow-up time

All surgeries were performed under general anesthesia using standard procedures. Meniscus tears were repaired first with either all-inside/inside-out or outside-in technique. All tendon grafts were then harvested from the hamstring tendon. Double-bundle ACL reconstruction was performed by the trans-tibia technique, aiming at the anatomic femoral footprint. Lastly, the femoral tunnel was fixed with the trans-tibial technique, followed by the tibia tunnel fixation.

### Clinical function evaluation

The functional outcomes were assessed with the, Tegner, International Knee Documentation Committee (IKDC) score and Lysholm score preoperatively and every years post-operatively for a minimum of 8 years. Pre-injury and post-operative (8 years post-operatively) UCLA activity scale were also collected in order to evaluate the degree of return to sports.

The patients were separated into two groups, lateral slope < 7.4° and lateral slope ≥ 7.4°, according to a previous report by Grassi [16]. The PROMS and demographic data were compared between the two groups. In addition, the patients were categorized into four groups, Excellent (95–100), Good (84–94), Fair (65–83) and Poor (< 65), based on the postoperative Lysholm score. One-way ANOVA was then used to determine the significant differences between the groups.

### Radiographic evaluation

**Fig. 1:**
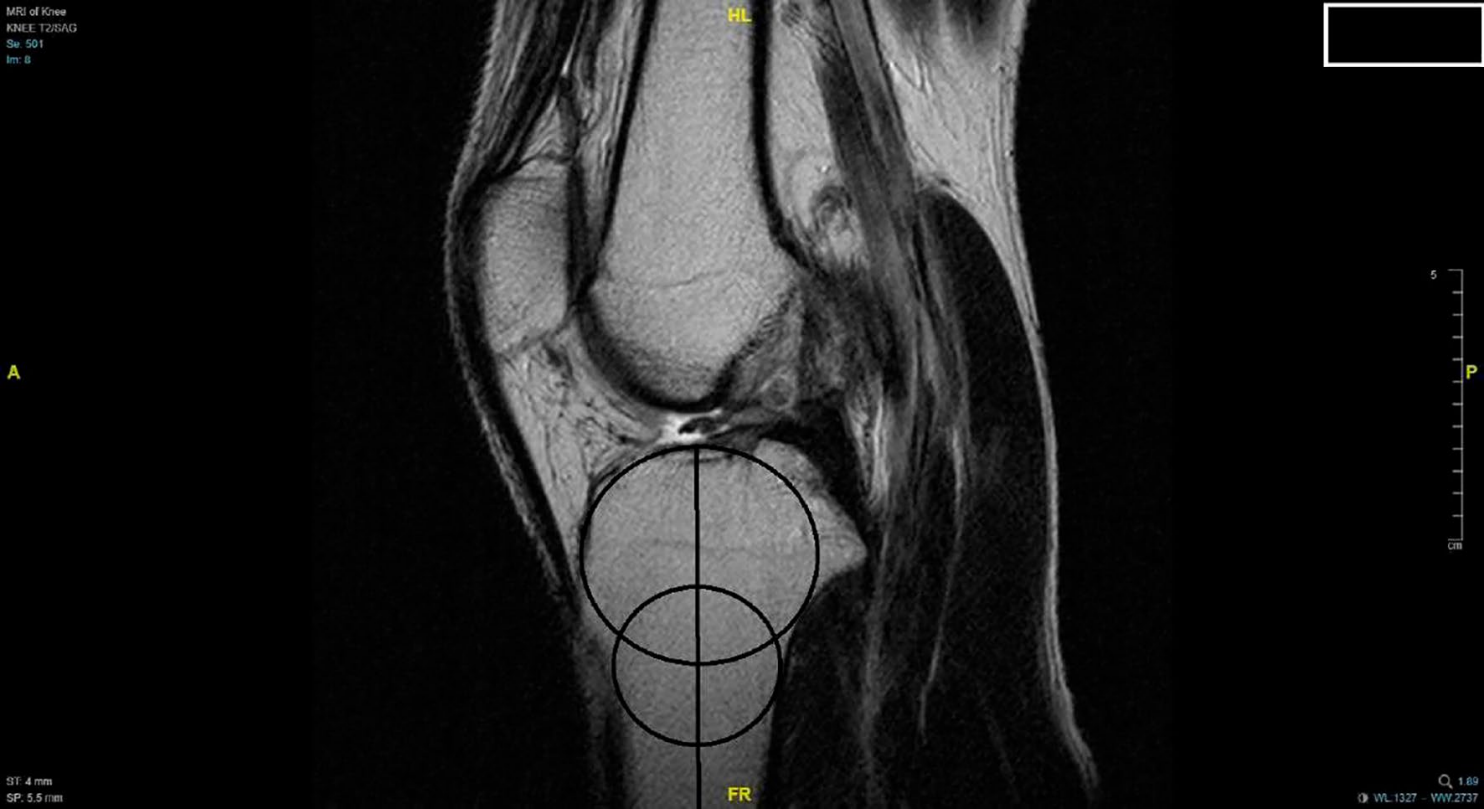
A 39-year-old male was diagnosed with torn ACL and received double-bundle ACL reconstruction. The anatomical tibial axis was measured by drawing two circles within the proximal tibia. The first circle was placed within the posterior cortical, proximal, and anterior borders of the tibia. The second circle was drawn within the posterior and anterior cortices with the center of the circle positioned on the perimeter of the first circle. A line that passes through the centers of both circles

**Fig. 2:**
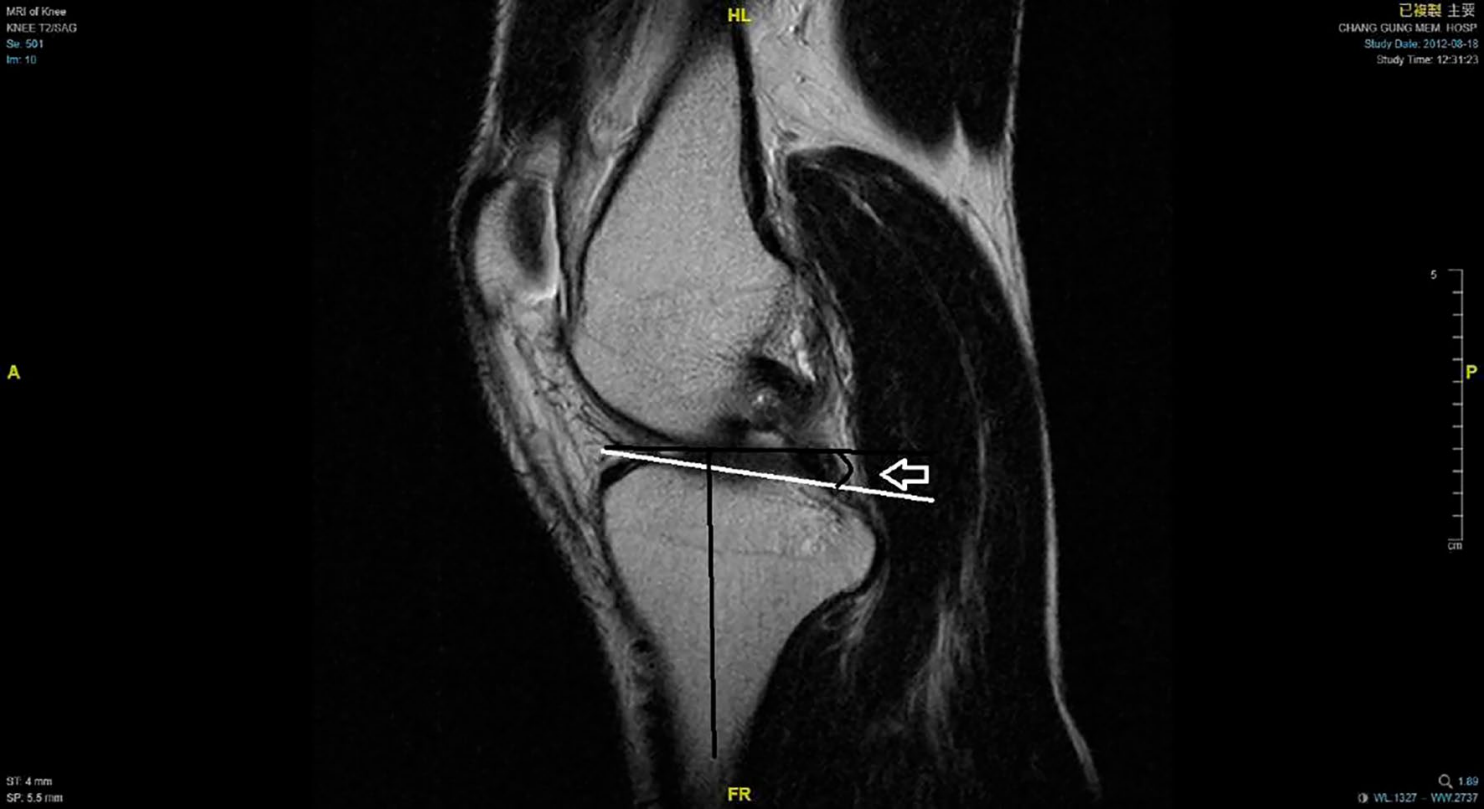
The medio-lateral center of the lateral plateau was then identified by a tangent to the uppermost even part between the superior anterior and posterior cortices. Lateral tibial slope was defined as the angle between the orthogonal to the proximal anatomical axis and the tangent to the lateral plateau

Using magnetic resonance imaging, lower limb alignment was measured and recorded by drawing two circles within the proximal tibia. The first circle was placed within the posterior cortical, proximal, and anterior borders of the tibia. The second circle was drawn within the posterior and anterior cortices with the center of the circle positioned on the perimeter of the first circle. A line that passes through the centers of both circles was defined as the lower limb alignment (Fig. 1). The medio-lateral center of the lateral plateau was then identified by a tangent to the uppermost even part between the superior anterior and posterior cortices. Lateral tibial slope was defined as the angle between the orthogonal to the proximal anatomical axis and the tangent to the lateral plateau (Fig. 2) [21]. Meniscus injury, collateral ligament integrity, and cartilage injury were also recorded using MRI. Radiographic evaluation was performed by an experienced surgeon and rechecked by the other two authors of this study that specialized in orthopedic knee surgery. These surgeon were blinded to the patient’s medical records. We also evaluated the inter-observer reliability of the slope measurement.

### Statistical analysis

Statistical Package was used for the Social Sciences (IBM SPSS Statistics 23.0) (IBM Inc., Armonk, NY, USA) and Microsoft Office Excel (Microsoft Office 2016). All categorical data were analyzed using Fisher’s exact test. The VAS score and IKDC and Lysholm scores were analyzed using the two-tailed Mann–Whitney *U* test. The results were considered statistically significant at a *p* value < 0.05. We used the Pearson *t* test for the correlation of medial/lateral slope and the patient-reported outcome measurements (PROMS).

## Results

**Fig. 3:**
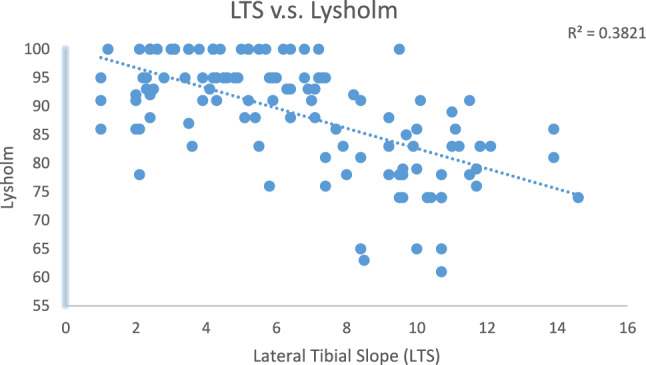
Linear correlation between lateral tibial slope and Lysholm Knee Scoring Scale

**Fig. 4:**
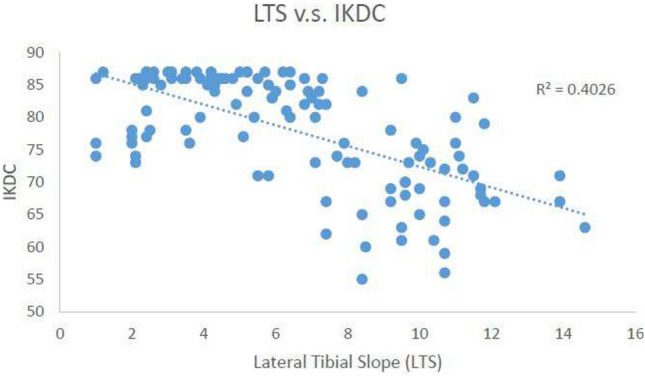
Linear correlation between lateral tibial slope and International Knee Documentation Committee Score

**Fig. 5:**
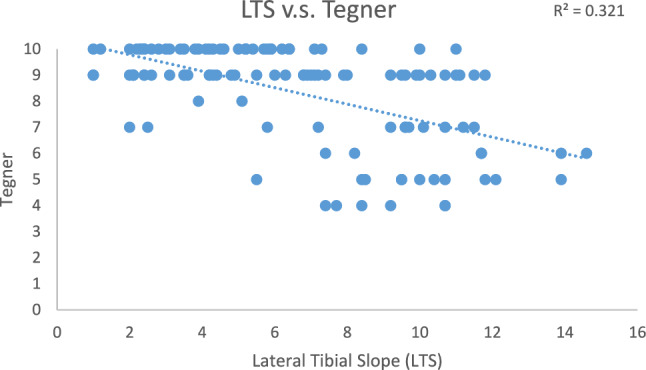
Linear correlation between lateral tibial slope and Tegner Activity Scale

Within the study group, 25 patients were excluded due to loss of follow-ups; 9 patients were excluded due to having previous knee surgery or revision; 1 patient was excluded due to malignant bone tumor. A total of 119 knees (119 patients) were reviewed. There were 113 males and 6 females. The mean age at surgery was 32.53 ± 7.61 years old and 44.33 ± 8.34 years old at the last follow-up. The mean BMI at surgery was 26.38 ± 4.26 (Table 1). The average lateral tibial slope and medial tibal slope were 6.56° ± 3.38° and 5.94° ± 4.37°, respectively. Sixty-seven of the patients suffered meniscus tear. A total of 11 patients had medial cartilage injury and 4 patients had lateral cartilage damage. Preoperatively, the UCLA activity was correlated with the lateral tibial slope. The mean pre-injury UCLA score was 8.33 ± 1.37. The average post-operative UCLA score was 7.14 ± 1.84 (Table 2). Postoperatively, the UCLA scale, Lysholm score, IKDC score and Tegner score were all negatively correlated with the lateral tibial slope (*p* values all < 0.001). On the other hand, the correlation was less significant in the medial tibial slope. The correlation analysis results are listed in Figs. 3, 4, and 5.

**Table 2:** UCLA activity scores

Pre-injury	8.33 ± 1.37	
Post-operative	7.14 ± 1.84	

Values are the means ± standard deviations

Pre-injury: before ACL injury

Post-operative: 5 years after surgery

The group with a higher lateral tibial slope (≥ 7.4°) had significantly lower values than the group with lower lateral tibial slope (< 7.4) (Lysholm 94.26 ± 5.61 vs. 80.15 ± 8.28, *p* = 0.013; IKDC 82.99 ± 4.55 vs. 70.09 ± 7.15, *p* = 0.003; Tegner 9.32 ± 0.95 vs. 6.85 ± 1.99, *p* < 0.001), as shown in Table 3. The inter-observer reliability was measured and the intraclass correlation coefficient (ICC) was 0.82 (*p* < 0.001), as listed in Table 4.

**Table 3:** Functional outcomes

	LTS < 7.4°	LTS ≥ 7.4°	*p* value
Numbers	72	47	
Lysholm	94.26 ± 5.61	80.15 ± 8.28	*p = 0.013
IKDC	82.99 ± 4.55	70.09 ± 7.15	*p = 0.003
Tegner	9.32 ± 0.95	6.85 ± 1.99	*p < 0.001

Values are the means ± standard deviations

*LTS* lateral tibial slope, *Lysholm* Lysholm Knee Scoring Scale, *IKDC* International Knee Documentation Committee Score, *Tegner* Tegner Activity Scale

^*^p < 0.05, statistical significance

**Table 4:** Interobserver reliability of measurement for tibial slopes

Interobserver reliability of measurement for tibial slopes			
	ICC	95% CI	*p* value
LTS on MRI	0.820	0.491–0.951	< 0.001

Values are the means ± standard deviations

*LTS* lateral tibial slope, *ICC* intraclass correlation coefficient

Using Lysholm score scale, there were 45 patients in the Excellent group, and the mean LTS was 4.46° ± 1.88°. Thirty six patients were in the Good group with the mean LTS of 5.98° ± 3.39°. The Fair group had 36 patients, and the mean LTS was 9.59° ± 2.53°. The Poor group had two patients, and the mean LTS was 9.60° ± 1.56°. The one way ANOVA showed significant difference between each groups. The cutoff value for the lateral tibial slope between the “good” and “fair” groups was 7.55° using receiver operating characteristic curve, which was close to the cutoff value from Grassi. The results are shown in Table 5.

**Table 5:** Lysholm score ranking

	Number (%)	LTS mean (degrees)
Excellent (95–100)	45 (37.8%)	4.46° ± 1.88°
Good (84–94)	36 (30.2%)	5.98° ± 3.39°
Fair (65–83)	36 (30.2%)	9.59° ± 2.53°
Poor (< 65)	2 (1.7%)	9.60° ± 1.56°

One way ANOVA, *p* < 0.001

When 2 patients from the Poor group were excluded, *p* remained < 0.001

^*^LTS cutoff between good and fair: 7.55°

During the follow-ups, 2 (a 40-year-old male and a 28-year-old female) out of the 119 patients underwent revision ACL reconstruction (6 months and 13 months post-operatively, respectively), and their LTSs were 8.0° and 8.4°, respectively. Both patients were heavily engaged in sport activity.

## Discussion

Our study showed that patients with higher LTS may experience inferior clinical outcomes after receiving DB ACL reconstruction. These patients had significantly lower PROMS values (Lysholm 94.26 ± 5.61 vs 80.15 ± 8.28, *p* = 0.013; IKDC 82.99 ± 4.55 vs 70.09 ± 7.15, *p* = 0.003; Tegner 9.32 ± 0.95 vs 6.85 ± 1.99, *p* < 0.001). Besides, the LTS cutoff value between patients with “Good” and “Fair” Lysholm score in our study was 7.55 degrees. This cut-off value is close to 7.4° which was suggested to possess higher risk of ACL failure after primary reconstruction [16]

Previous cadaveric studies have shown that the anatomy of the ACL is composed of anteromedial (AM) and posterolateral (PL) bundles [8, 40]. Due to the attempt to restore the anatomic structure, double-bundle ACL reconstruction has been one of the many methods used for treating ACL tears and knee instability. In some laboratory studies, double-bundle ACL reconstruction can better restore the stability of the knee compared to single-bundle ACL reconstruction. Double-bundle ACL reconstruction allows patients to have better control over their knee joint rotation [4, 9, 26, 28]. In previous long-term clinical results, double-bundle ACL reconstruction seemed to have less graft failure and residual anterior translation, though the subjective outcome seemed to be similar between the two groups in long-term follow-up [9, 10, 12, 19, 20, 33, 35]. In our study, only two out of 119 patients had failed ACL reconstruction after 8 years of follow-up.

There have been multiple studies that assess risk factors for ACL graft failure, including lateral tibial slope [2, 3, 15, 17, 23, 24, 30, 32, 36, 41]. Lachlan et al. concluded in their stability study that ligamentous laxity, male sex, posterior third medial or lateral meniscal injury, increased posterior tibial slope, and chronicity were related to a high-grade pivot shift and higher risk for repeated ACL injury [3]. Even though functional outcomes after ACL reconstruction are influenced by multiple factors, the role of the tibial slope in pivot-induced ACL injury has become more important in recent years, as several studies have disclosed [6, 13, 18, 24, 27, 31, 34]. Grassi et al. conducted a case‒control study in 2019 and enrolled 43 patients who experienced graft failure after primary ACL reconstruction [16]. While that study only mentioned the graft type (all hamstring tendon), the surgical method used was not recorded. Among several anatomical variables that were compared, the lateral tibial slopes of the knees were significantly different between the graft failure group and the control group. In our study, there was a strong negative correlation between lateral tibial slope and postoperative PROMS data.

The clinical effect of double-bundle ACL reconstruction has been debated for years [7, 9, 10, 12, 28, 33]. Jarvela et al. conducted a prospective randomized study of 90 patients that received ACL reconstruction with either double-bundle or single-bundle ACL reconstruction with bioabsorbable screw fixation and the graft failure rate was significantly higher in the single-bundle group [19]. The remaining 70 patients had no difference in the pivot shift test, arthrometer measurement, or knee scores. In addition, the rate of osteoarthritis was not different among the groups. In Bedi et al., the double-bundle ACL reconstruction was significantly better in limiting anterior translation of the lateral compartment compared to the single-bundle ACL reconstruction during a pivot-shift maneuver and was not significantly different than the intact anterior cruciate ligament condition [4]. On the other hand, single-bundle ACL reconstruction resulted in significantly greater translation of the lateral compartment than double-bundle reconstruction [13, 34]. The translation of the lateral compartment and knee pivot are highly correlated with the lateral tibial slope. However, Gobbi et al.’s result showed that double-bundle ACL reconstruction did not show superior patient reported outcome measure (PROMs) compared to that of single-bundle ACL reconstruction, although no data regarding to patients’ lateral tibial slope was given [14]. In our study, however, patients with lower lateral tibial slope had better functional scores compared to patients with higher lateral tibial slope. It is likely that double-bundle ACL reconstruction may indeed provide patients with lower lateral tibial slope better long-term functional outcome. While double-bundle ACL reconstruction along does provide better stability of knee compared to single-bundle ACL reconstruction, our study result demonstrated that the steep tibial slope was too much to overcome even with the use of double-bundle ACL reconstruction.

In recent years, primary ACL reconstruction combined with either lateral extra-articular tenodesis (LET) or anterolateral ligament (ALL) reconstruction has been advocated for patients with high risk of graft failure [25, 29]. The high-risk groups included adolescents and athletes participating high pivot sports. These techniques were known to provide better control for rotational stability and anterior translation. Our results suggested that patients with higher LTS may need either LET or ALL reconstruction in the primary reconstruction. However, further studies are necessary to evaluate the clinical effect of ACL reconstruction combined LET or ALL reconstruction in patients with high LTS.

Arthrometer examination can be useful in evaluating post-operative outcome of ACL-injured patients. While the worse functional outcomes in our study may be due to residual pain and stiffness rather than instability, the graft failure rate and knee laxity may be related to PROMS-related data. Lindanger et al. separated patients after ACL reconstruction with bone–patellar tendon–bone grafts into tight grafts (KT-1000 data 6 m after primary surgery: < 3-mm side-to-side difference) and slightly loose (3–5 mm) and loose grafts (> 5 mm) [1]. Patients with tight grafts tended to have higher Lysholm scores after 24 months and after 25 years, compared to 78% and 33% for patients with slightly loose grafts. Kaeding et al. reviewed data from the Multicenter Orthopedic Outcomes Network (MOON) and compared the ipsilateral knee graft retear rate along with age, sex, Marx activity score, graft type, lateral meniscus tear, medial meniscus tear, sport played at index injury, and surgical facility. [2] The results showed that the Marks activity score was related to the ipsilateral ACL retear rate. We believe that arthrometer data should be included in future studies.

### Limitation

There were some limitations in this study. First of all, there were no arthrometer measurements. Our institution did not apply arthrometer examination (GNRB) as part of our standard protocol for ACL-injured patients until after 2019. Secondly, male patients was dominated in our study group (113 males and 6 females), and the study population was not randomized, which might have led to selection bias. In addition, the radiographic measurement was done by one surgeon and rechecked by two other surgeons. The inter-observer reliability was inevitable. Lastly, there was a lack of objective return to sport information from some of the patients.

## Conclusions

Patients with high lateral tibial slope may result in inferior long term subjective outcomes. The using of double-bundle ACL reconstruction along cannot overcome the negative impact caused by steep lateral tibial slope. A lateral tibial slope of 7.55° may be used as a cut-off for a good clinical outcome.
